# The Relationship between Marital Status and Psychological Resilience in Chronic Pain

**DOI:** 10.1155/2013/928473

**Published:** 2013-09-15

**Authors:** James B. Wade, Robert P. Hart, James H. Wade, Jasmohan S. Bajaj, Donald D. Price

**Affiliations:** ^1^Department of Psychiatry, Virginia Commonwealth University, P.O. Box 980268, Richmond, VA 23298-0268, USA; ^2^Department of Psychology, Virginia Polytechnic University, 109 Williams Hall, Blacksburg, VA 24061, USA; ^3^Division of Gastroenterology, Hepatology and Nutrition, Virginia Commonwealth University, P.O. Box 980341, Richmond, VA 23298-0341, USA

## Abstract

We examined the relationship between marital status and a 2-stage model of pain-related effect, consisting of pain unpleasantness and suffering. We studied 1914 chronic pain patients using multivariate analysis of covariance (MANCOVA) to clarify whether marital status was a determinant factor in the emotional or ideational suffering associated with chronic pain after controlling for pain sensation intensity, age, and ethnicity. Marital status was unrelated to immediate unpleasantness (*P* = 0.08). We found a strong association with emotional suffering (*P* < 0.0001) but not with negative illness beliefs (*P* = 0.44). Interestingly, widowed subjects experienced significantly less frustration, fear, and anger than all other groups (married, divorced, separated, or single). A final MANCOVA including sex as a covariate revealed that the emotional response to pain was the same for both widow and widower. Only those individuals whose spouse died experienced less emotional turmoil in the face of a condition threatening their lifestyle. These data suggest that after experiencing the death of a spouse, an individual may derive some “emotional inoculation” against future lifestyle threat.

## 1. Introduction

High levels of happiness or well-being are associated with beneficial outcomes, such as healthy development of young adults [1] and longevity [2]. Research conducted internationally points to a strong association between level of social support and well-being [3]. Helliwell et al. [4] used data from the World Gallup Poll and found that the strength of an individual's social network determined their well-being. A study conducted in Seoul, South Korea [5], showed that individuals who had somebody to “lean on” in times of trouble reported higher well-being. In a South African study, those residents reporting the strongest feelings of well-being also enjoyed the greatest amount of social support from community members [6]. Conversely, in a study conducted in five countries throughout Asia, results indicated that lacking somebody to discuss important matters with was associated with lower well-being [7]. In a Belgian study [8], higher levels of life satisfaction were associated with strong social ties, even after adjusting for levels of optimism. In Germany, those people who reported a high frequency of visiting friends, relatives, or neighbors were almost 20% more satisfied with their lives [9]. These data suggest that the strength of social ties is associated with well-being and life satisfaction throughout the world. 

Marriage is associated with longer life and better health in both men and women [10]. Marriage connects people to other individuals, to social groups (e.g., extended family), and to other social institutions, which are additional sources of social benefit [11]. Based on the above evidence we anticipated that being married would serve as a buffer against the psychological suffering associated with chronic pain. Specifically, we hypothesized that chronic pain patients who were married would suffer less than individuals who were single, separated, divorced, or widowed. For over half a century pain has been conceptualized as a multidimensional construct, consisting of sensory-discriminative, cognitive-evaluative, and affective-motivational dimensions [12]. These dimensions have been further characterized as representing different stages of pain processing [13–15]. The first stage of pain processing is a sensory-discriminative dimension, which is associated with intensities and specific qualities of somatosensory sensations (e.g., “burning,” “stinging,” “aching,” etc.). The second stage of pain reflects an individual's moment by moment feelings such as unpleasantness, distress, and annoyance. Although it is associated with painful sensation, it involves limited cognitive processing and is often closely associated with the perceived intrusiveness of the painful sensation. As this stage invokes only limited cognitive appraisal, it is only modestly influenced by personality factors [13, 16]. The third stage of pain processing is cognitively mediated by an individual's beliefs, attitudes, and reflections concerning the real or imagined long-term consequences of having pain. This pain stage is the product of long-term cognitive or reflective processes that are related to the meanings or implications that pain holds for one's life [17]. The combined ratings of depression, anxiety, frustration, fear, and anger have been shown to represent this unique psychological component distinguishable from immediate pain-related unpleasantness [14, 16, 18]. This third stage of processing can be divided into an emotional and ideational realm. While a patient may deny significant levels of depression or anxiety associated with their pain, they may instead emphasize how difficult it is for them to endure pain over time. They may experience dramatic disruption of lifestyle, an inability to reduce the intensity of their pain, and a sense of helplessness or lack of control over their own health. 

To evaluate the influence of marital status on pain-related affect we separately measured both the immediate pain-related unpleasantness and the second stage of pain effect and suffering, using both emotional and ideational indicators. For there to be emotional differences in response to pain based on an individual's marital status, extensive processing involving reflection on past experience and an anticipation of pain's impact on the future would be necessary. Therefore, we hypothesized that, as opposed to pain-related unpleasantness, suffering would differ depending on an individual's marital status. Further, we anticipated married chronic pain sufferers to benefit from spousal support and therefore suffer less compared to the other marital groups. In addressing this question we studied a large sample of chronic pain sufferers prior to beginning treatment at a southeastern university-based pain center. Prior research has indicated differences in pain-related suffering by age [19] and ethnicity [20]. Therefore, in examining the relationship between marital status and well-being in chronic pain patients we statistically controlled for ethnicity and age, as well as pain sensation intensity.

## 2. Materials and Methods

### 2.1. Subjects

Subjects were patients seen at a Southeastern United States University medical center pain clinic between 1990 and 2000. During this time period a total of 1914 patients were evaluated at the pain center. The patient ages ranged from 16 to 73 years (mean = 41.7, SD = 9.39). One thousand two hundred and sixty-three patients (66%) were Caucasian, 593 patients (31%) were African-American, and 58 patients (3%) were Asian-Americans. There were 1038 women and 876 men. Subjects completed an average of 11.88 years of education (SD = 3.05). Two hundred and seventy-three patients (14%) were single, 1052 patients (55%) were married, 349 (18%) were divorced, 125 patients (7%) were separated, and 113 patients (6%) widowed. Pain duration was on average 1.82 years (SD = 0.97). The most frequent diagnosis was failed back syndrome (*N* = 609), followed by myofascial dysfunction (*N* = 336), and complex regional pain syndrome (*N* = 151).

### 2.2. Measures

Prior to beginning medical therapy, patients underwent a psychological test battery conducted by a clinical psychologist. Subjects completed the MCV Pain Questionnaire [13, 15, 21]. This questionnaire consisted of a series of Visual Analog Scales (VAS) scales 15 cm in length with verbal anchor points. For pain sensation intensity, the anchor points were “no sensation” and “the most intense sensation imaginable.” Subjects placed a mark reflecting their pain sensation at its highest, usual, and lowest intensity during the prior week. For pain unpleasantness the anchor points were “not bad at all” and “the most intense bad feeling imaginable.” Subjects placed a mark reflecting how disturbing their pain was at its highest, usual, and lowest unpleasantness during the previous week. The negative emotion VAS anchor points were “none” and “the most severe imaginable.” Specifically, we asked patients, “what kind of negative feelings accompany your pain? Place a mark along each scale below, indicating the intensity of each feeling as it is related to your pain problem over the past week.” With regard to the negative emotion VAS, the subject was asked to place a mark along each scale reflecting the intensity of their depression, anxiety, frustration, fear, and anger only as it pertained to their chronic pain. Four VAS were used to assess the subject's perception regarding the pain's impact on his/her life. Using the same 15-centimeter scale, subject's rated how difficult it was to endure pain over time, to what extent the pain interfered with their lives, how much they felt able to reduce the intensity of their pain, and how much control they felt they had over their own health. Ratings were obtained during a period without intervening treatment. These scales have been demonstrated to be reliable and valid measures of pain sensation intensity [13, 15] and pain-related negative emotions associated with pain [14]. The test-retest reliabilities of these VAS have been shown to be high, with values ranging from 0.70 to 0.90 [14]. Several studies support the reliability, validity, and sensitivity of VAS measures of emotion, either as single-item measures of specific emotions [14, 22–24] or as a composite measure [14, 16, 20, 21, 25]. 

Each subject was administered the Beck Depression Inventory-II [26] a widely used self-rating scale for the assessment of depression in adults and adolescents older than 13 years. The questionnaire consists of 21 groups of statements. A subject reads each of the group statements and selects one statement in each group that best describes how they have been feeling during the past two weeks. A total score is derived by adding the highest number (zero through 3) from each of the 21 groups. Higher total scores are associated with greater levels of depression. Validation studies have shown high test-retest reliability and internal consistency [27, 28] and moderate to high convergent and divergent validity [26, 29].

Subjects also completed the Social Support Scale of the Multidimensional Pain Inventory (MPI) [30]. The MPI is a 60-item self-report inventory that assesses a patient's psychosocial and behavioral responses to their chronic pain condition. The Social Support Scale is a 14-item empirically derived scale that requires the subject to read a sentence and indicate how often significantly others respond in a particular way. For example, following the sentence, “Asks me what he or she can do to help,” the subject circles a number between 0 and 6. Each scale has verbal anchor points labeled “never” and “very often.” A higher total score reflects a greater degree of interpersonal support. The MPI has been widely used with diverse samples of chronic and subacute pain patients (e.g., headache, back pain, temporomandibular disorders, cancer, fibromyalgia, and neck pain). Research examining the Support Scale has demonstrated good reliability and validity [31, 32], as well as sensitivity to change [33–37]. The Institutional Review Board at Virginia Commonwealth University approved the protocol.

### 2.3. Data Analysis

To clarify whether marital status is a determinant factor in emotional and/or ideational suffering associated with chronic pain, Multivariate Analysis of Covariance (MANCOVA) using SPSS statistical software version 21 [38] was conducted. In this multivariate general linear modeling procedure we statistically controlled for pain sensation at its highest, usual, and lowest intensity during the previous week, along with age and ethnicity. 

## 3. Results

A series of MANCOVA were conducted, the first of which revealed that after controlling for pain sensation intensity, age, and ethnicity, marital status was not related to stage 1 affect, or what we have termed immediate unpleasantness (*F* = 1.617, df = 12, *P* = .08). Two additional MANCOVA revealed that marital status was uniquely associated with stage 2 affect, what we have termed emotional suffering (*F* = 2.880, df = 20, *P* < .0001) but not negative illness beliefs (*F* = 1.012, df = 16, *P* = .440). 

Post hoc pairwise analyses, using Bonferroni correction to account for multiple comparisons, were used to identify differences between the marital groups in emotional suffering. Our hypothesis that married individuals would benefit from this close relationship and suffer the least emotional turmoil in response to pain received only limited support. As seen in Table 1, married subjects reported less depression than divorced individuals (mean difference = −.7, *P* < .03) and less anger than single individuals (mean difference = −.9, *P* < .008). Interestingly, pain patients who had experienced the death of a spouse (e.g., the widow group) suffered significantly less depression compared to those who were divorced or separated and less anxiety (Table 2) compared to those who were divorced or single. They also experienced less frustration (Table 3), anger (Table 4), and fear (Table 5) than all other groups (divorced, single, separated, or married). Similarly, Analysis of Covariance (ANCOVA) revealed that after controlling for pain sensation intensity, age, and ethnicity, marital status was related to depression severity, as measured by the Beck Depression Inventory-II (*F* = 18.323, df = 4, *P* < .001). Married subjects experienced significantly less depression compared to the divorced (mean difference = −2.8, *P* < .000) and separated (mean difference = −3.2, *P* < .006) subjects. Widowed subjects like married patients manifested significantly less depression compared to divorced (mean difference = −5.0, *P* < .000) and separated (mean difference = −5.4, *P* < .000) as well as the single (mean difference = −3.3, *P* < .023) subjects. Therefore, across both the MCV Pain Questionnaire and the Beck Depression Inventory, the widow/widower group demonstrated remarkable emotional resilience to chronic pain.

To clarify whether widows and widowers differed in their emotional response to pain, a final MANCOVA was conducted that included the term sex as a covariate (along with pain intensity, age, and ethnicity). The main effect for sex (*f* = 1.442, df = 5, *P* = .206) and the interaction of sex with marital status (*f* = .482, df = 20.0, *P* = .974) were not significant. Finally, ANCOVA was conducted to determine whether the differences seen in emotional suffering between marital groups were a function of interpersonal support as measured by the MPI. After controlling for pain sensation intensity, age, and ethnicity, the marital groups did not differ on this measure (*f* = .617, df = 4, *P* = .650). 

## 4. Discussion

Marital status was not associated with immediate pain-related unpleasantness. Our hypothesis that being married would be associated with lower levels of pain-related emotional suffering was only weakly supported. The unexpected finding was that in the face of a condition threatening their lifestyle (e.g., chronic pain) those subjects who had experienced the death of a spouse suffered significantly less frustration, fear, and anger than subjects in all other marital categories and less depression and anxiety than divorced individuals. Although separated and divorced subjects experienced the loss of a spousal relationship, only those subjects whose spouse had died experienced less emotional turmoil in response to pain. Importantly, the level of perceived social support and severity of self-perceptions of pain impact (e.g., ability to endure pain over time, perceived extent of lifestyle interference, ability to reduce the pain intensity, and perceived extent of control over their health) did not differ across the marital groups. Therefore, mitigation of emotional suffering by widow/widowers was not the result of this marital group enjoying greater social support or the belief that their pain resulted in less lifestyle disruption. 

For there to be emotional differences in response to pain higher level cognitive processes related to the consideration of future consequences and rumination concerning the past would seem necessary. Pain-related unpleasantness reflects an individual's immediate moment by moment responses to the painful sensation and involves limited cognitive processing [16]. Consistent with this notion immediate pain-related unpleasantness appears to be only modestly influenced by personality factors [13, 16, 21, 25]. For this reason it is not surprising that in our subjects immediate unpleasantness did not differ as a function of marital status. Suffering on the other hand is more cognitively mediated by an individual's beliefs, attitudes, and reflections on the real or imagined long-term consequences of having pain. Along these lines Leknes et al. [39] demonstrated that by altering the context of an experimental pain experience the same pain sensation intensity was viewed by subjects as either emotionally upsetting or a “pleasant relief.” This “hedonic flip” of the pain's meaning was corroborated by both physiological and functional neuroimaging data. 

Brain imaging studies suggest that unlike immediate unpleasantness, emotional suffering is associated with brain activation in regions associated with language processes, episodic memory, and executive function which serves to direct psychological operations to produce an emotional gestalt. Divorced and separated individuals differ from widows and widowers in that they were more active participants and presumably had more control over their change in marital status. Losing a spouse to illness appears to have a different impact on an individual than divorce or separation. Experiencing the death of a loved one may lead an individual to develop additional coping strategies, or in some other way, make them less vulnerable to emotional distress in reaction to future lifestyle threat. Given that lifetime expectancy in the United States is increasing, a spouse may survive many years following the death of their partner. The results of this study are encouraging for the surviving spouse's emotional resilience, at least for some types of future stressors. Our data suggest that after experiencing the trauma of the death of a spouse, an individual may derive some “emotional inoculation” against future lifestyle threat. This finding speaks to the strength of the human spirit to recover and to develop new psychological strength, after misfortune. Such psychological growth is not simply a product of wisdom that comes with age. The statistical control of age in the analysis did not weaken the relationship between widow/widower status and intensity of emotional turmoil in response to chronic pain. There appears to be no sex difference with regard to this form of emotional resilience. Also, this emotional buffer developed despite having no fewer negative illness beliefs, suggesting a dissociation between an individual's emotional resilience and ideational adjustment to life-changing events.

The process of “psychological stability” may provide a useful explanation for our main finding. Psychological flexibility involves interaction between the environment and cognition that allows an individual to persist, or change, in accordance with their values and long-term goals [40]. Component processes include mindfulness, value-based action, and acceptance. Prior research has demonstrated significant relationships between components of psychological flexibility and physical and emotional adaptation to chronic pain [41]. It is possible that loss of a spouse facilitates greater psychological flexibility, by virtue of being confronted by, and forced to adapt to, one of the life's greatest losses. Consistent with our findings, older age per se is not associated with higher levels of such psychological flexibility [42]. Future studies examining the relationship between marital status and pain-related suffering might assess measures of psychological flexibility, such as acceptance.

A limitation of the study is that we did not include measures assessing the broader social network. Prior research on pain and marital status [43], for example, suggests that a nondistressed marital relationship is associated with less pain and better functioning in rheumatoid arthritis patients. While being married was only marginally associated with less pain-related emotional suffering, the importance of the quality of the marital relationship should be explored in future studies. Similarly, future studies may wish to further assess other contributions to suffering within the family system (e.g., having to take care of an invalid relative, economic problems, etc.). In addition, careful interviewing of widows and widowers might clarify the nature of gained resilience accounting for better emotional well-being. It should be noted that the pain duration for study subjects was about 2 years. It is unclear whether the identified relationships between marital status and suffering would generalize to individuals with greater pain duration. 

## 5. Conclusions

In this study, after statistically controlling for pain sensation intensity, age, and ethnicity, marital status was uniquely associated with emotional suffering. Interestingly, compared to all other marital categories, in response to a condition threatening their lifestyle (e.g., chronic pain), those subjects that experienced the death of a spouse suffered less. Specifically, widowed subjects experienced significantly less frustration, fear, and anger than all other groups (married, divorced, separated, or single). Furthermore, the emotional response to pain was the same for both widow and widower. Although separated and divorced subjects experienced the loss of a spousal relationship, it was only those individuals whose spouse died that experienced less emotional turmoil in response to pain. These data suggest that after experiencing the death of a spouse, an individual may derive some “emotional inoculation” against future lifestyle threat. These data have important social implication. Given that lifetime expectancy in the United States is increasing, a spouse may survive many years following the death of their partner. The results of this study are encouraging for the surviving spouse's emotional resilience in the face of future lifestyle stressors.

## Figures and Tables

**Table 1:** Post hoc pairwise comparisons between depression VAS and marital status.

	Widow versus single	Married versus single	Separated versus single	Divorced versus single
Mean difference	−1.058	−.418	.437	.286
Standard error	.437	.264	.419	.315
Significance	.155	1.00	1.00	1.00

	Widow versus married	Married versus divorced	Separated versus divorced	

Mean difference	−.640	−.704	.152	
Standard error	.387	.241	.405	
Significance	.985	.035*	1.00	

	Widow versus divorced	Married versus separated		

Mean difference	−1.344	−.856		
Standard error	.423	.367		
Significance	.015*	.198		

	Widow versus separated			

Mean difference	−1.496			
Standard error	.505			
Significance	.031*			

Significance level obtained by the post hoc pairwise comparison *P ≤ .05, **P ≤ .01, ***P ≤ .001.

**Table 2:** Post hoc pairwise comparisons between anxiety VAS and marital status.

	Widow versus single	Married versus single	Separated versus single	Divorced versus single
Mean difference	−1.175	−.270	−.151	.335
Standard error	.425	.257	.408	.307
Significance	.058	1.00	1.00	1.00

	Widow versus married	Married versus divorced	Separated versus divorced	

Mean difference	−.906	−.604	−.486	
Standard error	.377	.235	.394	
Significance	.164	.101	1.00	

	Widow versus divorced	Married versus separated		

Mean difference	−1.510	−.119		
Standard error	.412	.358		
Significance	.003**	1.00		

	Widow versus separated			

Mean difference	−1.025			
Standard error	.492			
Significance	.375			

Significance level obtained by the post hoc pairwise comparison *P ≤ .05, **P ≤ .01, ***P ≤ .001.

**Table 3:** Post hoc pairwise comparisons between frustration VAS and marital status.

	Widow versus single	Married versus single	Separated versus single	Divorced versus single
Mean difference	−1.992	−.316	−.283	.198
Standard error	.414	.251	.398	.299
Significance	.000***	1.00	1.00	1.00

	Widow versus married	Married versus divorced	Separated versus divorced	

Mean difference	−1.676	−.514	−.481	
Standard error	.367	.228	.384	
Significance	.000***	.246	1.00	

	Widow versus divorced	Married versus separated		

Mean difference	−2.190	−.033		
Standard error	.401	.348		
Significance	.000***	1.00		

	Widow versus separated			

Mean difference	−1.709			
Standard error	.479			
Significance	.004**			

Significance level obtained by the post hoc pairwise comparison *P ≤ .05, **P ≤ .01, ***P ≤ .001.

**Table 4:** Post hoc pairwise comparisons between anger VAS and marital status.

	Widow versus single	Married versus single	Separated versus single	Divorced versus single
Mean difference	−2.337	−.954	−.330	−.548
Standard error	.468	.283	.449	.337
Significance	.000***	.008**	1.00	1.00

	Widow versus married	Married versus divorced	Separated versus divorced	

Mean difference	−1.383	−.405	.219	
Standard error	.414	.258	.434	
Significance	.009**	1.00	1.00	

	Widow versus divorced	Married versus separated		

Mean difference	−1.789	−.624		
Standard error	.453	.393		
Significance	.001**	1.00		

	Widow versus separated			

Mean difference	−2.007			
Standard error	.541			
Significance	.002**			

Significance level obtained by the post hoc pairwise comparison *P ≤ .05, **P ≤ .01, ***P ≤ .001.

**Table 5:** Post hoc pairwise comparisons between fear VAS and marital status.

	Widow versus single	Married versus single	Separated versus single	Divorced versus single
Mean difference	−2.164	−.598	.215	.077
standard error	.464	.281	.445	.334
Significance	.000***	.333	1.00	1.00

	Widow versus married	Married versus divorced	Separated versus divorced	

Mean difference	−1.566	−.675	.138	
Standard error	.411	.256	.430	
Significance	.001***	.084	1.00	

	Widow versus divorced	Married versus separated		

Mean difference	−2.241	−.813		
Standard error	.450	.390		
Significance	.000***	.372		

	Widow versus separated			

Mean difference	−2.379			
Standard error	.537			
Significance	.000***			

Significance level obtained by the post hoc pairwise comparison *P ≤ .05, **P ≤ .01, ***P ≤ .001.

## References

[1] Park N (2004). The role of subjective well-being in positive youth development. *Annals of the American Academy of Political and Social Science*.

[2] Diener E, Chan MY (2011). Happy people live longer: subjective-well-being contributes to health and longevity. *Applied Psychology*.

[3] Calvo R, Zheng Y, Kumar S, Olgiati T, Berkman L (2012). Well-being and social capital on planet Earth: cross-national evidence from 142 countries. *Proceedings of the Library of Science*.

[4] Helliwell JF, Barrington-Leigh C, Harris A, Huang H, Diener E, Helliwll JF, Kahneman D (2010). International evidence on the social context of well-being.

[5] Han S, Kim H, Lee H-S (2013). A multilevel analysis of the compositional and contextual association of social capital in subjective well-being in Seoul, South Korea. *Social Indicators Research*.

[6] Cramm JM, Møller V, Nieboer AP (2012). Individual and neighborhoods-level indicators of subjective-well being in a small and poor Eastern Cape township: the effects of health, social capital, marital status and income. *Social Indicators Research*.

[7] Yamaoka K (2008). Social capital and health and well-being in East Asia: a population-based study. *Social Science and Medicine*.

[8] Hooghe M, Vanhoutte B (2011). Subjective well-being and social capital in Belgian communities: the impact of community characteristics on subjective well-being indicators in Belgium. *Social Indicators Research*.

[9] Winkelmann R (2009). Unemployment, social capital, and subjective well-being. *Journal of Happiness Studies*.

[10] Waite LJ (1995). Does marriage matter?. *Demography*.

[11] Stolzenberg RMM, Blair-Loy TJ, Waite LJ (1995). Religious participation over the life course: age and family life cycle effects on church membership. *American Sociological Review*.

[12] Melzack R, Casey KL, Kenshalo DR (1968). Sensroy, motivational, and central control of determinatnts of pain. *The Skin Senses*.

[13] Harkins SW, Price DD, Braith J (1989). Effects of extraversion and neuroticism on experimental pain, clinical pain, and illness behavior. *Pain*.

[14] Wade JB, Price DD, Hamer RM, Schwartz SM, Hart RP (1990). An emotional component analysis of chronic pain. *Pain*.

[15] Price DD, McGrath PA, Rafii A, Buckingham B (1983). The validation of visual analogue scales as ratio scale measures for chronic and experimental pain. *Pain*.

[16] Wade JB, Riddle DL, Price DD, Dumenci L (2011). Role of pain catastrophizing during pain processing in a cohort of patients with chronic and severe arthritic knee pain. *Pain*.

[17] Price DD (2000). Psychological and neural mechanisms of the affective dimension of pain. *Science*.

[18] Wade JB, Price DD, Gatchel RJ, Weisberg JN (2000). Nonpathological factors in chronic pan: implications for assessment and treatment. *Personality Characteristics of Pain Patients*.

[19] Riley JL, Wade JB, Robinson ME, Price DD (2000). The stages of pain processing across the adult lifespan. *Journal of Pain*.

[20] Riley JL, Wade JB, Myers CD, Sheffield D, Papas RK, Price DD (2002). Racial/ethnic differences in the experience of chronic pain. *Pain*.

[21] Wade JB, Dougherty LM, Archer CR, Price DD (1996). Assessing the stages of pain processing: a multivariate analytical approach. *Pain*.

[22] Maruff P, Wood S, Currie J, McArthur-Jackson C, Malone V, Benson E (1994). Computer-administered visual analogue mood scales: rapid and valid assessment of mood in HIV positive individuals. *Psychological Reports*.

[23] Knight RG, Thirkettle JA (1987). Anxiety and depression in the immediate post-partum period: a controlled investigation of a primiparous sample. *Australian and New Zealand Journal of Psychiatry*.

[24] Millar K, Jelicic M, Bonke B, Asbury AJ (1995). Assessment of preoperative anxiety: comparison of measures in patients awaiting surgery for breast cancer. *British Journal of Anaesthesia*.

[25] Wade JB, Dougherty LM, Hart RP, Rafii A, Price DD (1992). A canonical correlation analysis of the influence of neuroticism and extraversion on chronic pain, suffering, and pain behavior. *Pain*.

[26] Beck AT, Steer RA, Brown GK (1996). *Manual for the Beck Depression Inventory-II*.

[27] Arnau RC, Meagher MW, Norris MP, Bramson R (2001). Psychometric evaluation of the Beck Depression Inventory-II with primary medical care patients. *Health Psychology*.

[28] Steer RA, Rissmiller DJ, Beck AT (2000). Use of the Beck Depression Inventory-II with depressed geriatric inpatients. *Behaviour Research and Therapy*.

[29] Kapci EG, Uslu R, Turkcapar H, Karaoglan A (2008). Beck depression inventory II: evaluation of the psychometric properties and cut-off points in a Turkish adult population. *Depression and Anxiety*.

[30] Turk DC, Rudy TE (1988). Toward an empirically derived taxonomy of chronic pain patients: integration of psychological assessment data. *Journal of Consulting and Clinical Psychology*.

[31] Kerns RD, Turk DC, Rudy TE (1985). The West Haven-Yale Multidimensional Pain Inventory (WHYMPI). *Pain*.

[32] Mikail SF, DuBreuil S, D’Eon JL (1993). A comparative analysis of measures used in the assessment of chronic pain patients. *Psychological Assessment*.

[33] Donceel P, Du Bois M (1999). Predictors for work incapacity continuing after disc surgery. *Scandinavian Journal of Work, Environment and Health*.

[34] Bernstein IH, Jaremko ME, Hinkley BS (1995). On the utility of the West Haven-Yale Multidimensional Pain Inventory. *Spine*.

[35] Flor H, Birbaumer N (1993). Comparison of the efficacy of electromyographic biofeedback, cognitive-behavioral therapy, and conservative medical interventions in the treatment of chronic musculoskeletal pain. *Journal of Consulting and Clinical Psychology*.

[36] Scharff L, Turk DC, Marcus DA (1995). Psychosocial and behavioral characteristics in chronic headache patients: support for a continuum and dual-diagnostic approach. *Cephalalgia*.

[37] Turk DC, Zaki HS, Rudy TE (1993). Effects of intraoral appliance and biofeedback/stress management alone and in combination in treating pain and depression in patients with temporomandibular disorders. *The Journal of Prosthetic Dentistry*.

[38] Statistical Package for the Social Sciences (SPSS).

[39] Leknes S, Berna C, Lee MC, Snyder GD, Biele G, Tracey I (2013). The importance of context: when relative relief renders pain pleasant. *Pain*.

[40] Hayes SC, Luoma JB, Bond FW, Masuda A, Lillis J (2006). Acceptance and commitment therapy: model, process and outcomes. *Behaviour Research and Therapy*.

[41] McCracken LM, Velleman SC (2010). Psychological flexibility in adults with chronic pain: a study of acceptance, mindfulness, and values-based action in primary care. *Pain*.

[42] Hayes SC, Strosahl K, Wilson KG (2004). Measuring experiential avoidance: a preliminary test of a working model. *Psychological Record*.

[43] Reese JB, Somers TJ, Keefe FJ, Mosley-Williams A, Lumley MA (2010). Pain and functioning of rheumatoid arthritis patients based on marital status: is a distressed marriage preferable to no marriage?. *Journal of Pain*.

